# Therapeutic applications of red marine seaweeds in dental care: innovations in oral health and treatment

**DOI:** 10.2340/biid.v12.45224

**Published:** 2025-12-29

**Authors:** Sivakamavalli Jeyachandran

**Affiliations:** Lab in Biotechnology and Biosignal Transduction, Department of Orthodontics, Saveetha Dental College and Hospital, Saveetha Institute of Medical and Technical Sciences (SIMATS), Saveetha University, Chennai, Tamil Nadu, India

**Keywords:** Red marine seaweeds, dental care, therapeutic applications, carrageenan, oral health, antimicrobial properties

## Abstract

Red marine seaweeds, particularly those belonging to the genus *Rhodophyta*, possess a robust structural framework and are rich in biologically active compounds such as carrageenan, agar, collagen, and alginate. These natural resources are abundant and have attracted significant attention for their therapeutic potential in oral healthcare. This review highlights the applications of red marine seaweeds in dentistry, focusing on their roles in maintaining oral health, facilitating wound healing, exhibiting antibacterial properties, and contributing to tooth remineralization. Notably, their anti-inflammatory activity supports the management of conditions such as gingivitis, periodontitis, and oral ulcerations, while their antibacterial and antifungal actions effectively inhibit oral pathogens such as *Streptococcus mutans*, a primary contributor to dental caries. Furthermore, carrageenan-based biodegradable films derived from red seaweeds demonstrate promising potential as controlled drug delivery systems and tissue-regenerative biomaterials within the oral cavity. Beyond clinical applications, seaweeds can also serve as sustainable sources for formulating naturally derived oral hygiene products – including mouthwashes, toothpastes, and gels – offering eco-friendly alternatives to synthetic chemicals. However, despite these advances, broader clinical adoption requires comprehensive and well-designed clinical trials to validate efficacy and safety. Overall, this review underscores the emerging potential of red marine seaweeds as sustainable, bioactive resources for innovative dental therapeutics, while emphasizing the need for continued translational and clinical research.

## Introduction

The growing demand for natural, sustainable, and biocompatible materials in dentistry has accelerated research into alternatives to conventional synthetic biomaterials. Among these, red marine seaweeds (*Rhodophyta*) have emerged as promising candidates due to their abundance, structural diversity, and wealth of bioactive compounds. Coastal ecosystems host over 7,000 species of red algae, many of which contain polysaccharides such as carrageenan, agar, and fucoidan, along with proteins and lipids that contribute to their biomedical potential (Ahmad et al., 2022; Carpena et al., 2023). These compounds exhibit antimicrobial, anti-inflammatory, and regenerative properties, making them attractive for applications in oral health and dental therapeutics (Liyanage et al., 2023). In dentistry, red seaweed-derived biomaterials show potential in multiple areas, including periodontal tissue regeneration, wound healing, oral biofilms inhibition, and caries prevention. Sulfated polysaccharides like carrageenan and fucoidan, in particular, have demonstrated strong antibacterial effects against oral pathogens such as *Streptococcus mutans*, as well as significant tissue-repairing capabilities (Dou et al., 2023; Thitame et al., 2025). Their natural biocompatibility and biodegradability further position them as eco-friendly alternatives to petroleum-based and chemically synthesized dental materials, aligning with the expanding market for sustainable dental products (Das & Bal, 2024; Martin & Mulligan, 2022).

This review aims to provide a comprehensive overview of the therapeutic potential of red marine seaweeds in dental care. It explores their role in antimicrobial defense, anti-inflammatory action, wound and periodontal tissue regeneration, as well as their integration into oral hygiene formulations such as toothpastes, mouthwashes, and gels. Particular attention is given to their applicability in developing biocompatible scaffolds, dental implants, and drug delivery systems. By consolidating current evidence, this work emphasizes the translational prospects of red seaweeds as innovative, sustainable resources for future dental therapeutics.

## Dentistry and the antimicrobial properties of red marine seaweeds

**Figure 1:**
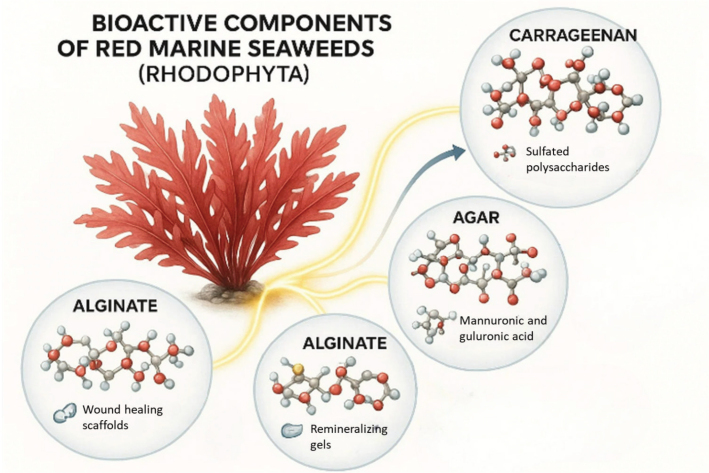
Bioactive Components of Red Marine Seaweeds (Rhodophyta). A schematic representation showing key compounds such as carrageenan, agar, and alginate extracted from red marine seaweeds, highlighting their structural forms and relevance in dental applications.

Red marine seaweeds of the phylum *Rhodophyta* are increasingly recognized for their potent antimicrobial and bioactive properties, which have promising applications in dentistry. They are rich in sulfated polysaccharides such as carrageenan, agar, and fucoidan, along with proteins, phenolic compounds, and other metabolites that contribute to their antimicrobial, antifungal, and anti-inflammatory activities (Figure 1). These bioactive molecules are of particular interest in oral healthcare, as they can suppress oral pathogens, disrupt biofilms, and reduce inflammation, thereby supporting both preventive and therapeutic dental strategies. Several red seaweed species have been studied in the context of dentistry. For example, *Kappaphycus alvarezii* and *Eucheuma denticulatum* are major commercial sources of carrageenan, which has demonstrated antibacterial and antiviral properties relevant to oral infections. *Gracilaria verrucosa* and *Gelidium amansii*, rich in agar, have shown potential in biomedical formulations due to their gelling properties and ability to deliver antimicrobial agents. Species such as *Chondrus crispus* (Irish moss) and *Hypnea musciformis* are also reported to contain sulfated galactans with inhibitory effects on bacterial adhesion and plaque formation (Marunganathan et al., 2024; Tabassum et al., 2023).

Fucoidan, although classically associated with brown seaweeds, has also been isolated from certain red algal species such as *Gymnogongrus griffithsiae* and *Nemalion helminthoides*. It acts as a sulfated fucan with strong antibacterial and antibiofilm properties. Its mechanism of action involves increasing bacterial cell membrane permeability, leading to cell lysis, as well as disrupting the ability of oral pathogens such as *S. mutans* to form biofilms – thereby reducing the risk of dental caries and periodontitis (Sun et al., 2022). Similarly, carrageenan exhibits antimicrobial activity by inhibiting bacterial adhesion to oral tissues and limiting the initiation of biofilms. In addition, carrageenan has been linked to immunomodulatory effects, which further enhance host defense mechanisms in the oral cavity (Marunganathan et al., 2024). Taken together, the antimicrobial properties of red marine seaweeds and their species-specific bioactive compounds highlight their potential role in dental applications. By targeting both microbial growth and biofilm formation, these natural polysaccharides offer an eco-friendly and biocompatible alternative to synthetic antimicrobials in the development of oral care products such as toothpastes, mouthwashes, gels, and biodegradable films (Table 1).

**Table 1:** Red marine seaweeds in dental applications: Antibacterial and antibiofilm activity.

Seaweed Species/Compound	Bioactive Compound(s)	Antibacterial Activity	Antibiofilm Activity	References
*Chondrus crispus*	Carrageenan	Inhibits *Streptococcus* mutans, *Porphyromonas* gingivalis, *Lactobacillus* species; strong antibacterial action against oral pathogens.	Disrupts biofilm formation of *Streptococcus mutans* and *Lactobacillus* species.	Rodríguez-Jasso et al., 2021; Zhao et al., 2021
*Gracilaria spp.*	Agar	Exhibits antimicrobial activity against *Streptococcus mutans*, *Actinomyces* species, and *Lactobacillus*.	Inhibits *Streptococcus mutans* adhesion and biofilm formation.	Rodríguez-Jasso et al., 2021
*Fucus vesiculosus*	Fucoidan	Inhibits *Streptococcus mutans*, *Porphyromonas gingivalis*, and *Fusobacterium nucleatum*.	Reduces biofilm formation of *Porphyromonas gingivalis* and *Streptococcus mutans*.	He et al., 2020; Zhao et al., 2021
*Eucheuma denticulatum*	Carrageenan	Effective against *Streptococcus mutans*, *Porphyromonas gingivalis*, and *Prevotella intermedia*.	Inhibits plaque-forming ability of *Streptococcus mutans* and other oral pathogens.	He et al., 2020
*Saccharina latissima*	Fucoidan	Exhibits antimicrobial properties against oral bacteria, particularly *Streptococcus mutans*.	Inhibits biofilm formation by *Streptococcus mutans* and *Lactobacillus species*.	Jung et al., 2022
*Palmaria palmata*	Carrageenan, Agar	Demonstrates significant antimicrobial effects on *Streptococcus mutans* and *Enterococcus faecalis*.	Reduces biofilm formation by *Streptococcus mutans*, helping to control dental plaque.	Sadiq et al., 2022
*Gelidium amansii*	Agar	Strong antimicrobial action against *Streptococcus mutans* and *Fusobacterium nucleatum*.	Prevents biofilm formation of *Streptococcus mutans*, potentially useful in preventing caries.	Le et al., 2020
*Corallina officinalis*	Carrageenan, Agar	Exhibits antibacterial activity against *Streptococcus mutans* and *Lactobacillus acidophilus*.	Inhibits biofilm formation, showing potential in preventing plaque buildup.	Zhou et al., 2021
*Kappaphycus alvarezii*	Carrageenan	Shows inhibitory effects on *Streptococcus mutans* and Porphyromonas gingivalis.	Reduces the formation of biofilms in oral bacteria, reducing plaque formation.	Rodrigues et al., 2021

### Continent effectiveness of oral pathogens

Bioactive compounds from red marine seaweeds have demonstrated significant antimicrobial activity against major oral pathogens, particularly *S. mutans* and *P. gingivalis*, which are primary contributors to dental caries and periodontal diseases, respectively. Sulfated polysaccharides such as fucoidan and carrageenan are the most studied in this regard. Fucoidan has been shown to inhibit bacterial adhesion and disrupt biofilm formation, thereby limiting the ability of *S. mutans* to colonize enamel surfaces and initiate caries development (Alfinaikh et al., 2025; He et al., 2020). In addition, fucoidan effectively reduces the virulence of *P. gingivalis*, impairing its capacity to attach to gingival tissues and decreasing the inflammatory response that drives periodontal pathology. Specific red seaweed species have been investigated for their role in oral pathogen control. *K. alvarezii* and *E. denticulatum* are widely utilized for carrageenan extraction, and their derivatives have demonstrated inhibitory activity against *S. mutans* biofilm formation. Extracts from *G. amansii* and *G. verrucosa*, rich in agar and sulfated galactans, have been tested in vitro and shown to reduce oral microbial load when incorporated into dental gels and rinses. Moreover, *C. crispus* and *H. musciformis* have yielded polysaccharide fractions that interfere with bacterial adhesion and promote a reduction in plaque accumulation.

**Figure 2:**
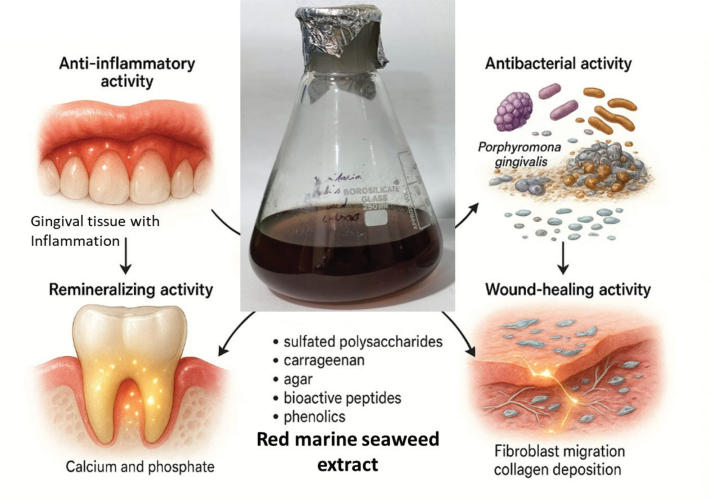
Mechanisms of Action of Red Seaweed-Derived Biopolymers in Oral Health. Illustration depicting how red seaweed components exhibit anti-inflammatory, antibacterial, remineralizing, and wound-healing activities in the oral cavity.

Recent studies have explored the integration of red seaweed extracts into oral care formulations. For instance, mouthwashes and toothpastes supplemented with carrageenan or fucoidan were shown to significantly decrease the bacterial burden of *S. mutans* and *P. gingivalis*, while also preventing plaque growth and improving overall oral hygiene (Zhao et al., 2024). These findings support the potential application of red seaweed-derived compounds as natural antimicrobial agents in preventive dentistry. Taken together, evidence indicates that red marine seaweeds, through their sulfated polysaccharides, can effectively target critical oral pathogens. By inhibiting adhesion, disrupting biofilms, and reducing microbial virulence, species such as *K. alvarezii*, *E. denticulatum*, *G. verrucosa*, *G. amansii*, *C. crispus*, and *H. musciformis* represent valuable resources for developing sustainable and biocompatible oral care products aimed at controlling caries and periodontal diseases (Figure 2) (Table 2).

**Table 2:** Mechanisms of action and applications of red marine seaweeds in dentistry.

Applications	Red Marine Seaweeds Used	Mechanisms of Action	Effectiveness/Results	References
Tissue Engineering Scaffolds	Carrageenan, Fucoidan, Agar	Mimics extracellular matrix; supports cell attachment, proliferation, and differentiation.	Promotes cell attachment and differentiation; biocompatible and biodegradable.	Rodríguez-Jasso et al., 2021; Zhao et al., 2021
Bone Regeneration (Bone Grafting)	Carrageenan, Hydroxyapatite	Provides structural integrity; supports osteoblast growth and osseointegration.	Enhanced bone formation; stimulates osteoblast proliferation.	Jung et al., 2022; Zhao et al., 2021
Periodontal Regeneration	Fucoidan	Promotes angiogenesis, reduces inflammation, supports periodontal ligament and bone regeneration.	Accelerates tissue repair, enhances fibroblast and osteoblast differentiation.	He et al., 2020; Zhao et al., 2021
Wound Healing in Periodontal Tissue	Fucoidan	Stimulates collagen formation, ECM deposition, and angiogenesis.	Reduces inflammation and promotes healing of periodontal defects and gingival wounds.	He et al., 2020; Rodríguez-Jasso et al., 2021

ECM: extracellular matrix.

## Plaque control and prevention potential caries

**Figure 3:**
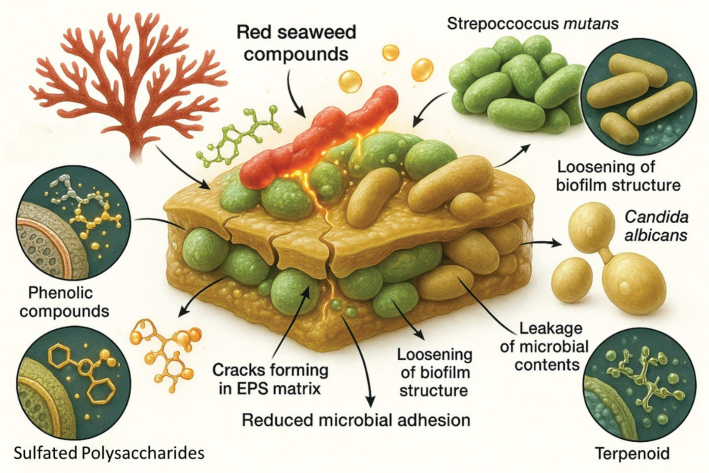
Antibacterial Effects of Red Marine Seaweed Compounds Against Oral Pathogens. Bar graph or heatmap showing inhibition zones or MIC values of red seaweed extracts against common oral pathogens such as Streptococcus mutans, Porphyromonas gingivalis, and Candida albicans.

One of the most significant advances in dental therapeutics has been the exploration of natural marine-derived compounds for the prevention of plaque formation and dental caries. Red marine seaweeds, particularly members of *Rhodophyta*, have shown strong potential in this context due to their bioactive polysaccharides, mainly fucoidan and carrageenan. Unlike conventional plaque-control agents such as triclosan and fluoride which, despite their efficacy, have raised concerns regarding long-term toxicity and environmental impact – seaweed-derived compounds present an eco-friendly, biocompatible, and sustainable alternative (Balasubramaniam et al., 2022). Mechanistically, fucoidan and carrageenan interfere with bacterial adhesion and biofilm development on tooth surfaces, thereby reducing the ability of *S. mutans* and other cariogenic bacteria to establish persistent colonies (Jung et al., 2022). By lowering bacterial load and inhibiting aggregation, these compounds prevent plaque maturation and limit acid production, which are critical steps in caries initiation. Furthermore, their immunomodulatory properties help suppress excessive inflammatory responses in the oral cavity, contributing to a balanced microbial environment and improved periodontal health (Figure 3).

Species-specific studies reinforce these observations. For example, extracts from *E. denticulatum* and *K. alvarezii*, rich in carrageenan, have demonstrated the ability to reduce biofilm density and plaque accumulation in vitro. Similarly, *C. crispus* and *H. musciformis* yield sulfated galactans with documented anti-plaque activity. The gelling and stabilizing properties of *G. verrucosa* make them particularly suitable for incorporation into oral care formulations such as gels, mouthwashes, and sustained-release dental films. Taken together, evidence suggests that red marine seaweeds hold considerable promise as natural plaque-control agents. Their dual role in reducing microbial burden and modulating host responses offers a sustainable approach to caries prevention and oral hygiene maintenance. By replacing or complementing synthetic chemicals, seaweed-derived compounds represent a forward-looking solution for safe and environmentally responsible dental care.

## Dentistry regenerative potential of red marine seaweeds

Red marine seaweeds, predominantly belonging to the phylum *Rhodophyta*, are increasingly recognized as valuable sources for regenerative applications in dentistry. Their unique biochemical composition, particularly the abundance of sulfated polysaccharides such as carrageenan, fucoidan, and agar, provides significant advantages for tissue repair and regeneration. Beyond their well-documented antimicrobial and anti-inflammatory properties, these compounds demonstrate excellent biocompatibility, making them suitable candidates for scaffold development in tissue engineering technologies. In regenerative dentistry, the bioactive constituents of red seaweeds contribute to multiple stages of the healing process. They have been reported to facilitate cellular adhesion, promote proliferation and differentiation, and support the deposition of extracellular matrix (ECM) components, all of which are critical for bone and periodontal tissue regeneration (Devi et al., 2022).

**Figure 4:**
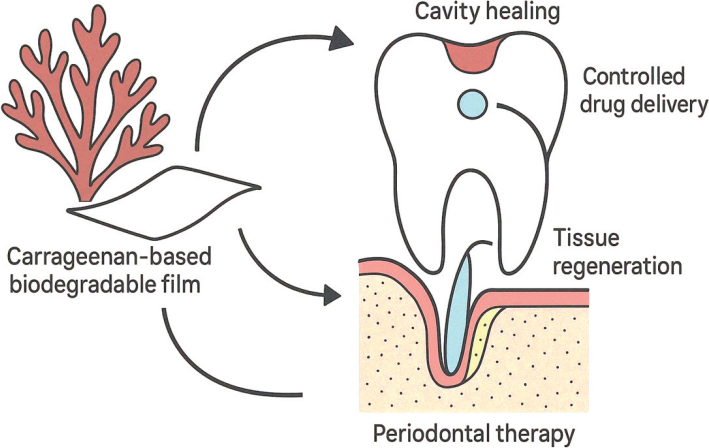
Red Marine Seaweed in Dental Regenerative Therapies. Diagram showing the potential application of carrageenan-based biodegradable films for controlled drug delivery and tissue regeneration in periodontal therapy and cavity healing.

Furthermore, the gelling and structural properties of seaweed-derived polysaccharides enhance their utility as biomaterials for designing hydrogels, films, and scaffolds that mimic the native extracellular environment. These materials not only provide mechanical stability but also actively participate in signaling pathways that guide tissue remodeling and repair in oral tissues, including alveolar bone and gingival structures. Taken together, the regenerative potential of red marine seaweeds highlights their role as sustainable, multifunctional biomaterials with applications in periodontal therapy, bone regeneration, and restorative dentistry. Their ability to integrate antimicrobial protection with regenerative capacity positions them as promising alternatives to conventional synthetic scaffolds in advancing dental tissue engineering (Figure 4).

### Red seaweed: A tissue-engineering scaffold

Scaffold development is a cornerstone of tissue engineering in dentistry, as it provides the three-dimensional framework required for cell adhesion, proliferation, and differentiation – processes essential for regenerating bone and periodontal tissues. Red marine seaweeds are emerging as promising candidates for scaffold fabrication, largely due to their rich content of sulfated polysaccharides such as carrageenan, fucoidan, and agar. These compounds closely mimic the structure and function of the natural ECM, thereby creating a biologically favorable environment for osteoblasts, fibroblasts, and stem cells involved in oral tissue repair (Kikionis et al., 2023). Several species of red seaweeds have demonstrated potential in this context. Extracts from *Porphyra yezoensis* are known to contain bioactive peptides and polysaccharides that promote osteoblast activity, suggesting possible utility in bone-regeneration scaffolds. *Palmaria palmata* has been investigated for its high protein and polysaccharide content, which supports cell adhesion and may enhance the regenerative capacity of periodontal scaffolds. Similarly, *Laurencia obtusa* produces halogenated secondary metabolites and sulfated polysaccharides with both antimicrobial and regenerative properties, making it a candidate for multifunctional scaffolding materials. In addition, *Acanthophora spicifera* has shown promise in wound-healing studies due to its sulfated galactans, which may be translated into dental tissue regeneration applications.

Importantly, carrageenan- and fucoidan-based scaffolds derived from these species not only provide structural integrity but also biodegrade at controlled rates, synchronizing scaffold resorption with new tissue growth and thereby reducing risks of chronic inflammation. Functional modifications of these polysaccharides further allow tailoring of bioactivity, including controlled release of bioactive molecules and enhanced cellular signaling. For example, fucoidan isolated from *Laurencia* spp. has been reported to stimulate mesenchymal stem cell differentiation into osteoblasts and chondrocytes, supporting alveolar bone remodeling and cartilage regeneration (Devi et al., 2022). Likewise, agar extracted from *Porphyra* and *Palmaria* species has been tested in hydrogel formulations that support fibroblast proliferation and periodontal tissue repair. These findings highlight the regenerative potential of diverse red marine seaweed species beyond the commonly studied genera. Their biochemical diversity, structural resemblance to the ECM, and ability to support cellular functions make them highly attractive for next-generation scaffolds in dental tissue engineering.

### Bone regeneration

Bone repair is a critical component of dental implantation and bone grafting procedures, where the integration of implants with surrounding bone tissue is essential for long-term success. Red marine seaweed-derived scaffolds, particularly those based on carrageenan, have attracted considerable attention for their role in bone regeneration due to their structural strength, biocompatibility, and resemblance to the natural ECM. These scaffolds provide mechanical stability while simultaneously supporting the biological processes of osteogenesis and osseointegration. Carrageenan-based scaffolds have been shown to promote the adhesion, proliferation, and differentiation of osteoblasts, which are key to new bone formation (Cao et al., 2022). When combined with bioactive compounds such as hydroxyapatite (HA), carrageenan scaffolds can mimic the mineralized bone matrix, thereby enhancing their potential for bone grafting and dental implant applications (Ocampo et al., 2019). Experimental studies have demonstrated that such scaffolds facilitate osteoblast-like cell growth, bone matrix deposition, and the direct bonding of implants to bone through osseointegration (Cao et al., 2021).

Beyond carrageenan, other red seaweeds also provide valuable biomaterials for bone regeneration. For instance, extracts from *G. corticata* have been investigated for their agar-derived hydrogels, which can serve as carriers for growth factors to stimulate osteogenesis (Sharmila et al., 2020). Similarly, sulfated polysaccharides from *G. amansii* have demonstrated osteoinductive properties by enhancing alkaline phosphatase activity and mineralized matrix formation in osteoblast-like cells (Lee et al., 2017). *L. papillosa*, another rhodophyte, has been reported to contain halogenated metabolites that not only exhibit antimicrobial activity but also enhance the biocompatibility of composite scaffolds used in bone tissue engineering (Ali et al., 2021). Fucoidan-containing composites from species such as *A. spicifera* have been found to modulate signaling pathways associated with bone differentiation, while polysaccharides from *P. tenera* have shown potential in promoting bone mineralization through antioxidant and osteoprotective effects (Kim et al., 2018). Importantly, the biodegradability of these scaffolds ensures that they are gradually resorbed and replaced by natural bone tissue, eliminating the need for surgical removal and reducing the risk of chronic inflammation. Altogether, these findings highlight the potential of red marine seaweed-derived scaffolds as multifunctional biomaterials in regenerative dentistry. By combining mechanical stability with bioactivity and biodegradability, they represent a promising alternative to conventional synthetic materials for bone grafting and implant integration.

### Periodontal regeneration

Periodontal diseases, particularly periodontitis, result in the progressive destruction of gingival tissue, periodontal ligament, and alveolar bone, ultimately leading to tooth loss if untreated. Red marine seaweed–derived biomolecules, particularly fucoidan and carrageenan, have emerged as promising candidates in periodontal regeneration due to their combined anti-inflammatory, angiogenic, and wound-healing properties. Fucoidan, a sulfated polysaccharide extracted from species such as *A. spicifera* and *Grateloupia filicina*, has demonstrated significant potential in periodontal tissue repair. Its mechanism involves modulation of inflammatory signaling pathways and stimulation of angiogenesis, which collectively promote periodontal ligament and alveolar bone regeneration (Eshwar et al., 2020). Studies indicate that fucoidan enhances fibroblast and osteoblast migration and differentiation, thereby accelerating the repair of damaged periodontal structures (Shim et al., 2022). Importantly, its role in facilitating collagen synthesis and ECM deposition contributes to both soft tissue healing and bone regeneration, providing a dual benefit in periodontal therapy (Xie et al., 2024).

Fucoidan’s regenerative potential is further supported by its ability to stimulate vascularization and fibroblast recruitment, which are essential for functional tissue restoration (Jeong et al., 2025). In vivo studies on fucoidan-containing hydrogels have demonstrated enhanced closure of periodontal defects, improved gingival wound healing, and restoration of structural integrity of the periodontium. Carrageenan, derived from species such as *K. alvarezii* and *E. denticulatum*, also plays an important role in periodontal regeneration. Carrageenan-based scaffolds create a three-dimensional network that provides fibroblasts and epithelial cells with a supportive environment for attachment, proliferation, and ECM deposition (Kikionis et al., 2023). This property accelerates gingival tissue repair and aids in the re-establishment of periodontal attachment. Recent experimental evidence suggests that carrageenan scaffolds can reduce local inflammation while promoting epithelial regeneration, making them suitable for incorporation into guided tissue regeneration membranes (Bajpai et al., 2024).

Additionally, agar-derived materials from *G. acerosa* have shown promise as biocompatible wound dressings with hemostatic and regenerative properties, which may be extended to periodontal applications. Their ability to maintain moisture and support cell migration makes them an attractive complementary material for periodontal tissue engineering. Together these findings establish red seaweed–derived polysaccharides as multifunctional biomaterials capable of supporting both hard and soft tissue regeneration in periodontal therapy. Their biocompatibility, bioactivity, and biodegradability highlight their potential as sustainable alternatives to synthetic scaffolds in modern dental regenerative approaches.

## Dental materials based on biocompatible red marine seaweed

In recent years, increasing attention has been directed toward the development of biocompatible and environmentally friendly dental materials. This trend reflects concerns over the long-term safety of synthetic polymers and their adverse ecological impacts. Red marine seaweeds (phylum *Rhodophyta*) have emerged as valuable sources of bioactive polysaccharides with the potential to serve as sustainable alternatives in dental applications. Among them, alginate, agar, and carrageenan are of particular significance due to their favorable physicochemical and biological properties. Alginate, widely used in impression materials, can be derived from species such as *G. edulis* and *G. dura*. Its gelling properties, dimensional stability, and biocompatibility make it a reliable material for impressions and casts. Agar, extracted from species such as *G. acerosa* and *G. pusillum*, is noted for its thermo-reversible gelation, high accuracy, and stability in reproducing fine details, which makes it suitable for use in prosthodontics and model preparation. Carrageenan, primarily obtained from *E. denticulatum* and *K. striatum*, offers mechanical strength, film-forming ability, and intrinsic antimicrobial activity. These properties support its application in dental composites, restorative fillings, and as a reinforcing agent in polymeric and ceramic blends.

The advantages of these seaweed-derived biomaterials extend beyond functionality. They are biodegradable, biocompatible, and capable of exerting antimicrobial activity, thereby reducing the risks of adverse tissue responses while simultaneously minimizing environmental toxicity. Importantly, their mechanical performance can be tailored when combined with polymeric or ceramic matrices, providing enhanced durability, microbial resistance, and long-term stability under functional loading (Jumaidin et al., 2018; Rambe et al., 2018). Collectively, their unique combination of biocompatibility, mechanical strength, and ecological sustainability underscores their potential in the development of next-generation dental biomaterials.

### Invention of seaweed-based dental products

Polysaccharide-rich red marine seaweeds are increasingly recognized as valuable resources for developing innovative dental materials due to their biocompatibility, natural origin, and capacity to support tissue healing. Among the most important polysaccharides derived from red algae are alginate, agar, and carrageenan, each of which has distinct advantages in dental applications. Alginate, obtained from species such as *G. edulis* and *G. dura*, is widely used in dental impression materials. It is favored for its ease of manipulation, short setting time, and ability to provide accurate impressions with minimal patient discomfort. Agar, extracted from *G. acerosa* and *G. pusillum*, is employed in impression materials due to its thermo-reversible gelation, high fidelity in detail reproduction, and cost-effectiveness. Its gel-forming ability allows it to compete with synthetic polymers in precision applications such as prosthodontics. Carrageenan, sourced primarily from *E. denticulatum* and *K. striatum*, has shown promise in more advanced applications, including dental fillings, crowns, and composite resins. Its adhesive properties, mechanical resilience, and natural antimicrobial potential make it suitable for blending with synthetic polymers to create multifunctional biomaterials (Buschmann & Sudhakar, 2024; Jumaidin et al., 2018).

**Figure 5:**
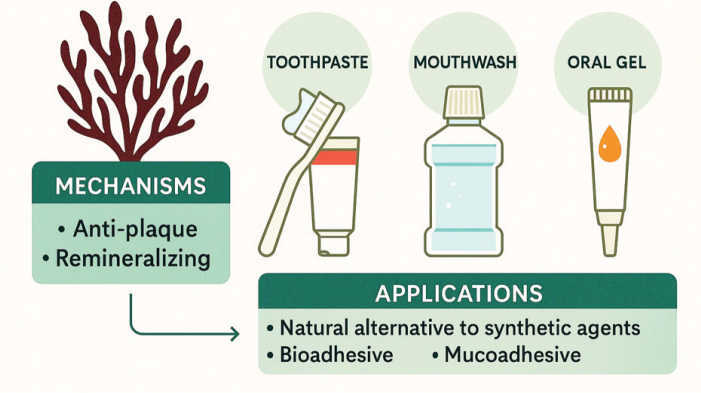
Natural Oral Care Products Formulated with Red Seaweed Extracts. Image montage or infographic highlighting formulations of toothpaste, mouthwash, and oral gels using red seaweed derivatives as natural alternatives to synthetic agents.

Recent research has focused on developing seaweed-based polymeric composites, which combine the biological advantages of marine polysaccharides with the mechanical strength of synthetic materials. Such composites are being tested for use in restorative dentistry, particularly in fillings and crowns, where they can mimic the hardness and elasticity of natural tooth structures while providing superior biocompatibility and reduced cytotoxicity (Sudhakar et al., 2023). In addition, their biodegradable nature provides an environmental advantage, offering a sustainable alternative to conventional petroleum-derived dental materials. In a recent study, carrageenan-based nanocomposites reinforced with HA were fabricated and evaluated for crown and filling applications. The material demonstrated comparable compressive strength to conventional resin-based composites while showing superior biocompatibility in fibroblast cultures and reduced bacterial adhesion against *S. mutans* (Buschmann & Sudhakar, 2024). These findings underscore the potential of carrageenan composites to serve as eco-friendly substitutes in restorative dentistry without compromising mechanical or antimicrobial performance (Figure 5).

## Biocompatibility and strengths

The potential of red marine seaweed–based dental materials lies primarily in their biocompatibility, biodegradability, and mechanical adaptability. These properties are critical for their integration into clinical dentistry, where safety, environmental sustainability, and durability are paramount. Seaweed-derived polysaccharides, including fucoidan, carrageenan, alginate, and agar, are naturally non-toxic, antimicrobial, and biodegradable, positioning them as attractive alternatives to synthetic polymers that often pose ecological and biocompatibility concerns. Importantly, their degradability ensures reduced long-term environmental impact compared to petroleum-based dental materials. Beyond safety, the inherent antimicrobial activities of fucoidan and carrageenan further enhance their value. These compounds inhibit bacterial adhesion and biofilm formation, reducing the risk of caries, gingivitis, and periodontitis when incorporated into dental composites and restorative materials (Akshayaa & Ganesh, 2025). Thus, seaweed-based materials combine structural and biological functions in ways that synthetic polymers often lack.

A central research challenge has been the mechanical performance of seaweed-derived biomaterials. On their own, these polysaccharides tend to have lower compressive strength and hardness compared to conventional resin composites. However, recent studies have focused on improving their properties through composite blending strategies. For instance, alginate reinforced with HA has shown improved compressive strength, hardness, and abrasion resistance while enhancing osteoconductivity – an essential property for dental implants and bone-regenerative applications (Huang et al., 2022). In addition, combining seaweed-derived polymers with synthetic biodegradable matrices such as polylactic acid (PLA) or polyethylene glycol (PEG) has produced composites with enhanced elasticity, tensile strength, and long-term durability. These hybrid materials provide resistance to wear and mechanical loading while maintaining the natural biocompatibility and biodegradability of marine polysaccharides (Sudhakar et al., 2023). Such approaches are gradually bridging the gap between natural marine-derived polymers and the performance requirements of clinical dental restoratives. Overall, while seaweed-based materials are still under development, the integration of biocompatibility, antimicrobial protection, and tunable mechanical strength makes them highly promising for future dental applications, including fillings, crowns, implants, and scaffolds.

### Coatings on dental implants and bioactive substances

The development of red marine seaweed–based materials as coatings for dental implants represents one of the most promising areas of innovation in modern dentistry. Implant success depends not only on mechanical stability but also on the ability of the implant surface to support biological integration with surrounding tissues. Seaweed-derived polysaccharides, particularly fucoidan and carrageenan, offer the dual benefits of biocompatibility and bioactivity, making them ideal candidates for functional implant coatings. Fucoidan, a sulfated polysaccharide isolated from species such as *A. spicifera* and *G. filicina*, has been shown to promote osteoblast differentiation, enhance bone formation, and stimulate angiogenesis – processes critical for the osseointegration of dental implants (Jin et al., 2023; Wen et al., 2023). By improving vascularization at the implant site, fucoidan accelerates tissue healing and reduces the risk of implant failure due to poor integration or infection.

Carrageenan, primarily obtained from *E. denticulatum*, has also demonstrated potential as a bioactive implant coating. Carrageenan-based surfaces improve osteoblast adhesion and proliferation, while simultaneously providing anti-inflammatory and wound-healing benefits during post-surgical recovery (Ocampo et al., 2019). These bioactive coatings mimic natural ECM properties, creating a surface that supports both structural stability and biological activity. The incorporation of seaweed-derived bioactive substances into implant coatings represents an alternative to conventional synthetic materials, which are often inert and lack biological interaction. Unlike many synthetic coatings, seaweed-based materials actively contribute to the healing process by modulating inflammation, reducing bacterial adhesion, and stimulating ECM deposition. This ensures not only faster healing but also long-term functional integration of the implant with surrounding bone and gingival tissues. Overall, coatings derived from red marine seaweeds combine biocompatibility, antimicrobial activity, osteoconductivity, and angiogenesis stimulation, offering a multifunctional approach that could significantly improve the long-term success of dental implants and reduce complications such as infection and implant rejection.

## Sustainability of red marine seaweed-derived materials and environmental impact

With increasing global concerns about environmental degradation and resource depletion, industries across multiple sectors are actively seeking sustainable and eco-friendly alternatives to traditional synthetic materials. Dentistry, in particular, faces growing scrutiny due to the environmental burden associated with the production, use, and disposal of conventional petrochemical-derived dental polymers and composites. These synthetic materials are often non-biodegradable, contribute to plastic waste, and may release toxic by-products into ecosystems over time. Red marine seaweeds have emerged as a promising sustainable alternative, offering biodegradability, renewability, and biocompatibility. Polysaccharides such as alginate, agar, and carrageenan – extracted from species like *G. edulis*, *G. acerosa*, and *E. denticulatum* – are being investigated as substitutes for petroleum-based polymers in dental materials. Unlike conventional plastics, which may persist in the environment for decades, seaweed-derived biopolymers degrade naturally, reducing the risk of long-term ecological damage.

A key difference lies in toxicity and patient safety. Traditional dental composites based on materials such as polymethyl methacrylate (PMMA) and bisphenol A-glycidyl methacrylate (Bis-GMA) can leach residual monomers, which are associated with cytotoxicity and potential systemic effects. By contrast, red seaweed-derived polysaccharides are inherently non-toxic and biocompatible, offering a safer alternative for both patients and practitioners. In terms of mechanical strength, synthetic composites still outperform seaweed-derived materials in load-bearing applications. However, advances in blending strategies – such as combining alginate or carrageenan with HA, PLA, or PEG – have significantly improved their compressive strength, abrasion resistance, and long-term durability, making them more competitive with conventional composites. Importantly, these blends maintain the antimicrobial and regenerative properties of the natural polysaccharides, features absent in conventional materials.

From a sustainability perspective, the advantages are even more pronounced. Red seaweed aquaculture is a renewable, low-carbon activity that can be scaled economically, while petrochemical-derived polymers are tied to fossil fuel consumption and greenhouse gas emissions. Thus, the adoption of red marine seaweed–based dental materials offers a dual benefit: reducing the ecological footprint of dentistry while simultaneously improving clinical safety and performance. Together, these comparisons demonstrate that while conventional synthetic materials remain strong and reliable, red seaweed–derived biopolymers present a sustainable, biocompatible, and multifunctional alternative that aligns with the goals of green dentistry and environmentally responsible innovation.

### Eco friendly and sustainable alternatives

Rising ecological concerns surrounding the reliance on petroleum-derived materials are driving dentistry toward the adoption of biodegradable and sustainable alternatives. Red marine seaweeds represent a particularly attractive resource in this transition. Their polysaccharides – alginate, agar, and carrageenan – are naturally biodegradable, ensuring that dental materials derived from them decompose safely after disposal. Unlike conventional synthetic polymers, which persist in the environment and contribute to landfill accumulation and microplastic pollution, seaweed-based biomaterials break down naturally, thereby minimizing ecological impact. One of the greatest advantages of seaweed-derived materials is their renewability. Red seaweeds are among the fastest-growing marine organisms, requiring no soil, pesticides, or freshwater, in contrast to the resource-intensive production of petrochemical-based plastics. This makes them not only renewable but also highly sustainable in large-scale applications.

Additionally, seaweed cultivation has a relatively low carbon footprint, as harvesting and processing are far less energy-intensive compared to petroleum refining and polymer synthesis (Ayala et al., 2024). The use of seaweed polysaccharides also aligns with the principles of green chemistry and safe material science. They are inherently biocompatible and non-toxic, reducing the risk of harmful leachates that are often associated with synthetic dental resins. This makes them particularly well-suited for applications where patient safety is paramount. For example, fucoidan, carrageenan, and agar exhibit low cytotoxicity and excellent compatibility with oral tissues, making them ideal candidates for dental impressions, restorative fillings, crowns, and even bioactive coatings on implants (Alli et al., 2022). In summary, red marine seaweed–derived dental materials provide a sustainable, eco-friendly alternative to conventional synthetic polymers. Their biodegradability, low environmental footprint, and biocompatibility highlight their potential to meet both ecological and clinical needs, offering a future where dental innovations are aligned with environmental responsibility.

### Minimizing reliance on petrochemical-based plastics in dental care products

The dental industry has traditionally relied on petrochemically derived plastics and synthetic composites, which contribute significantly to environmental pollution and carbon emissions (Mittal et al., 2020). These materials are durable and effective for clinical use but are non-biodegradable, resource-intensive, and environmentally damaging over their entire lifecycle. As global concerns about plastic waste intensify, dentistry faces increasing pressure to transition toward renewable and eco-friendly alternatives. Red marine seaweed–derived biomaterials present a sustainable solution to this challenge. Polysaccharides such as alginate and carrageenan, extracted from species like *G. edulis* and *E. denticulatum*, are already utilized in dental products including impression materials, restorative fillings, and crown composites. Compared to petrochemical-based polymers, these natural compounds are biocompatible, biodegradable, and renewable, offering both clinical safety and reduced ecological footprint (Putri et al., 2025).

Integrating seaweed-based biomaterials into dentistry also aligns with the principles of a circular economy, where materials are designed to be renewable, reusable, and biodegradable. This shift not only contributes to reducing plastic pollution but also ensures that dental products exert minimal long-term impact on ecosystems (Ayala et al., 2024). Furthermore, the scalability of seaweed cultivation provides a reliable raw material supply that reduces dependence on non-renewable petrochemical sources, thereby increasing self-sufficiency within the dental industry. Adopting seaweed-derived polymers as substitutes for petroleum-based plastics can therefore help the dental sector significantly lower its environmental footprint. Beyond the ecological benefits, such a transition would enhance the sustainability profile of dental materials across their entire lifecycle, from production and use to safe biodegradation after disposal. This positions red seaweed biomaterials as a key innovation in advancing green dentistry and addressing the global challenge of plastic pollution.

## Challenges and constraints to clinical use of red marine seaweeds

Despite their promise as sustainable, biocompatible, and regenerative dental materials, red marine seaweed–derived products face significant challenges in transitioning from laboratory research to clinical practice. A major limitation lies in scaling and processing. Extraction and purification of polysaccharides such as carrageenan, alginate, and fucoidan remain labor-intensive, requiring substantial water, energy, and time. Batch-to-batch variability in seaweed composition complicates consistency, while high production costs compared to petrochemical-derived polymers limit large-scale adoption (Chudasama et al., 2021; Nor et al., 2020). Furthermore, while these polysaccharides exhibit excellent biocompatibility, their mechanical properties often fall short of the high tensile strength, abrasion resistance, and durability required for dental restorations, necessitating reinforcement with synthetic polymers or inorganic fillers to achieve clinical-grade performance (Mirza et al., 2020).

Another critical barrier is the lack of robust clinical validation. Most evidence to date derives from in vitro or preclinical animal studies demonstrating antimicrobial, wound-healing, and tissue-regenerative effects (Alli et al., 2022). However, data on long-term clinical performance remain scarce. The oral cavity presents unique challenges – mechanical stresses from chewing and bruxism, fluctuating temperatures, and complex microbial communities – that demand durable materials. Biodegradability, while environmentally beneficial, raises concerns about material longevity and stability under these harsh conditions (González-Gloria et al., 2021). The absence of large-scale human clinical trials limits understanding of safety, efficacy, and durability in real-world dental applications (Sudhakar et al., 2023). In summary, the translation of red marine seaweed–derived materials into clinical dentistry is constrained by processing inefficiencies, mechanical limitations, and insufficient clinical evidence. Overcoming these barriers will require advances in scalable extraction, material reinforcement strategies, and extensive human clinical trials to establish their long-term viability as sustainable dental biomaterials.

## Research opportunities and future directions

Red marine seaweed–derived materials hold remarkable potential in dentistry, but further research is needed to overcome current limitations and fully unlock their clinical applications. A key challenge remains the mechanical enhancement of polysaccharides such as carrageenan, fucoidan, and alginate, which, despite their proven antimicrobial, anti-inflammatory, and regenerative properties, still lack the tensile strength, abrasion resistance, and durability required for long-term use in the oral cavity (Carrilho & Bretz, 2023). To address this, the development of hybrid composites combining seaweed polysaccharides with synthetic polymers, ceramics, or nanomaterials offers a promising strategy. Reinforcement with fillers such as HA or bioactive glass could improve osteoconductivity while maintaining biodegradability. Another important direction is the biotechnological optimization of seaweed resources. Genetic engineering and selective breeding of seaweed strains may help enhance the yield and bioactivity of key polysaccharides, producing more reliable raw materials with superior wound-healing and bone-regenerative potential (Kajla et al., 2024). Such advances would not only reduce variability in material properties but also improve cost-effectiveness and scalability.

In parallel, the future of dentistry is moving toward personalized and patient-specific treatments, and red seaweed-derived biomaterials are well-positioned to contribute. Customized scaffolds and composites tailored for individuals with bone defects, periodontal disease, or unique implant requirements could be designed by adjusting properties such as porosity, bioactivity, and mechanical strength. Emerging technologies such as 3D bioprinting and nano-functionalization offer exciting opportunities for fabricating made-to-order dental products that closely match patient anatomy and accelerate tissue healing (Waghmere et al., 2024). Taken together, future research must focus on enhancing mechanical performance, scaling production, and integrating personalization technologies to establish red marine seaweeds as clinically viable, sustainable, and customizable biomaterials. Their integration into regenerative and restorative dentistry could transform the field, combining ecological responsibility with advanced therapeutic outcomes.

## Conclusion

The exploration of red marine seaweed–derived materials in dentistry demonstrates a significant paradigm shift toward sustainable, biocompatible, and regenerative solutions. Throughout this article, the diverse applications of polysaccharides such as alginate, agar, carrageenan, and fucoidan have been highlighted, ranging from antimicrobial agents and plaque-control agents to scaffolds for bone and periodontal regeneration, bioactive implant coatings, and eco-friendly dental composites. Their inherent biodegradability, non-toxicity, and antimicrobial properties position them as attractive alternatives to conventional synthetic polymers, which are often associated with environmental hazards and potential cytotoxicity. Despite their promise, current challenges – such as the scaling of production, variability in chemical composition, limited mechanical strength, and the lack of robust clinical evidence – still hinder their full integration into mainstream dentistry. Addressing these barriers requires material reinforcement strategies, cost-efficient extraction technologies, and comprehensive clinical trials to validate their long-term safety, durability, and efficacy in the complex oral environment.

Looking forward, emerging technologies offer exciting avenues to enhance the performance and applicability of seaweed-based biomaterials. Nano-engineering approaches can significantly improve bioactivity, enabling closer interaction with cellular surfaces to stimulate adhesion, proliferation, and ECM deposition at implant sites (Zhang et al., 2021). Similarly, 3D bioprinting technologies incorporating seaweed-derived bioinks open the door to truly personalized dental solutions, allowing the fabrication of patient-specific scaffolds, implants, and restorations with optimized mechanical and biological properties. These innovations not only improve clinical precision but also support the transition toward green and sustainable dentistry by reducing reliance on petrochemical-based polymers. In conclusion, red marine seaweeds represent more than just a natural resource – they embody the convergence of marine biotechnology, material science, and clinical dentistry. With continued research to overcome current limitations and harness advanced technologies, they hold the potential to transform dental care by providing safer, eco-friendly, and regenerative biomaterials. Their integration into dentistry will not only enhance patient outcomes but also contribute to a sustainable healthcare system aligned with global environmental goals.
